# Retrospective analysis of machine service logs and EPID‐based linac QA to investigate the effects of machine maintenance and calibration events on beam characteristics: A multi‐institutional study

**DOI:** 10.1002/acm2.70894

**Published:** 2026-10-29

**Authors:** John DeMarco, Michael Barnes, Hania Al‐Hallaq, Seng Boh Lim, Lana Critchfield, Cory S Knill, Karl Farrey, Timothy Ritter, Nilendu Gupta, Richard A Popple, Justin Mikell, Jim Irrer, Mario Perez, Jean M Moran

**Affiliations:** ^1^ Cedars‐Sinai Department of Radiation Oncology NT Lower‐Level Los Angeles California USA; ^2^ Department of Radiation Oncology Calvary Mater Hospital Newcastle Waratah New South Wales Australia; ^3^ Department of Radiation Oncology Emory University Atlanta Georgia USA; ^4^ Memorial Sloan‐Kettering Cancer Center Radiation Dosimetry Core New York New York USA; ^5^ Department of Radiation Oncology University of Michigan Wyoming Michigan USA; ^6^ Department of Radiation Oncology University of Michigan Ann Arbor Michigan USA; ^7^ Radiation Oncology University of Chicago Chicago Illinois USA; ^8^ VCU Health System Radiation Oncology ‐ Adult Outpatient Pavilion (AOP) Richmond Virginia USA; ^9^ Department of Radiation Oncology The Ohio State University Columbus Ohio USA; ^10^ University of Alabama Birmingham Birmingham Alabama USA; ^11^ Department of Radiation Oncology Washington University St. Louis Missouri USA; ^12^ Northern Sydney LHD St Leonards (Sydney) Australia; ^13^ Memorial Sloan Kettering Cancer Center New York New York USA

**Keywords:** EPID, linear accelerator, quality assurance

## Abstract

**Background:**

Electronic Portal Imaging Devices (EPIDs) are widely used for LINAC quality assurance (QA) and underpin many manufacturer integrated quality control (MIQC) tools, yet the practical impact of routine linac service and calibration events on EPID‐derived beam characteristics remains under‐described. The Automated Quality Assurance (AQA) consortium has developed an independent, standardized EPID workflow for QA assessment that has been deployed across multiple TrueBeam linacs. We leveraged the AQA's framework and data to time‐align vendor service logs with automated EPID analyses and characterize event‐linked changes in photon beam output, symmetry, and collimator‐jaw positioning.

**Purpose:**

To investigate how EPID‐derived beam performance metrics change following common real‐world service events, including beam output calibration, active beam steering, and collimator jaw position calibration.

**Methods:**

We analyzed a multi‐institutional dataset acquired between 2018 and 2024 across nine TrueBeam LINACs using the AQA Phase‐2 EPID suite. EPID acquisitions were delivered in MV dosimetry mode and uploaded to a web portal for automated analysis with corresponding XML service logs to align service‐event timelines with EPID QA results. Service events were grouped into beam‐generation (dose calibration and active beam steering) and collimator (jaw position calibration) categories. The vendor XML service logs categorized events using labels such as “ANGLE R/T” or “POSITION servo” for beam‐steering adjustments and “GAIN‐OFFSET” for jaw position calibration. The EPID analysis included center‐dose constancy, axial and transverse beam symmetry, collimator‐centering correction, and X‐/Y‐jaw position. Measurement outliers were flagged using a density‐based spatial clustering of applications with noise (DBSCAN) algorithm applied separately to one‐dimensional EPID time series.

**Results:**

Across seven linacs with documented 6 MV dose calibrations, EPID center‐dose constancy showed a consistent post‐calibration decrease relative to baseline (mean ± SD = −1.10% ± 0.26%; range: −0.73% to −1.54%). In a paired subset (two linacs; *n* =  12 calibration events), the difference between EPID‐derived and ADCL‐calibrated ion‐chamber percent dose changes (ΔEPID − ΔIC) had a mean bias of 0.09% ± 0.79% (range −1.49% to 1.23%). Applying DBSCAN to the change in center‐dose output identified outlier points that were associated with lower (negative) changes across all nine linacs. Following ANGLE R/T steering, EPID‐derived symmetry shifts were observed and were concordant with daily detector‐array readings in a representative case, while symmetry changes also occurred after non‐steering activities (e.g., carousel/ion‐chamber work), indicating multiple service‐linked pathways affecting profile constancy. DBSCAN applied to jaw‐position metrics flagged outliers exceeding routine variability (e.g., 0.44 mm vs 0.03 mm), including a documented 1.3 mm Y2 shift temporally linked to a jaw GAIN‐OFFSET calibration.

**Conclusions:**

Integrating service logs with automated EPID analysis enabled event‐level interpretation of longitudinal QA measurements. EPID center‐dose changes following 6 MV dose calibrations were independently compared with ADCL‐traceable ion‐chamber measurements for a limited subset of two linacs, whereas the symmetry, jaw‐position, and DBSCAN findings represent EPID‐internal associations with documented service events and were not independently verified. Standardized EPID calibration records and prospectively timed pre‐ and post‐service measurements are needed to strengthen attribution and confirm the broader applicability of these findings.

## 1 INTRODUCTION

Electronic Portal Imaging Devices (EPIDs) have become a commonly used detector for linear‐accelerator (LINAC) quality assurance (QA) and have been extensively studied as tools for relative dosimetry and geometric verification in routine QA programs ^1^, ^2^, ^3^, ^4^, ^5^, ^6^. Prior work has demonstrated EPID capability for monitoring photon output and beam profile constancy, profiling and assessing dosimetric stability including sensitivity to subtle output variations that could impact treatment accuracy ^7^, ^8^, ^9^. EPIDs have also enabled automated QA workflows that streamline delivery and analysis, and manufacturer integrated quality control (MIQC) tools such as Machine Performance Check (MPC) have been validated against conventional measurements and intentional perturbations ^10^, ^11^, ^12^, ^13^, ^14^. What remains less well described is the effect of real‐world service and calibration events on EPID‐measured beam characteristics as encountered in day‐to‐day operations. Linear accelerators are routinely monitored and may require adjustments to output, beam steering, and calibration of the collimation subsystems; however, the expected direction, magnitude, and variability of the post‐service EPID signatures and their timing relative to recorded maintenance are not comprehensively documented. Pearson et al. have previously demonstrated the ability of the EPID to detect true out of tolerance QA results using the vendor‐integrated MPC tool.^15^ In contrast, our study leverages the work of the Automated Quality Assurance Consortium (AQA), which developed web‐based software and a test suite based on a standardized EPID workflow with manufacturer‐independent analytics, enabling multi‐institution comparisons and an analysis of time‐aligned EPID QA results with vendor machine service logs. Eckhause et al. previously demonstrated that a fully automated AQA consortium developed EPID workflow could capture gantry, jaw, MLC, and output deviations well within TG‐142 tolerances while reducing hands‐on time to < 15 min per machine for the evaluated tests.^16^ Subsequent consortium publications verified the portability of using this test‐suite across multiple TrueBeam linacs and highlighted the value of pooling results for cross‐site benchmarking with an automated EPID test for leaf‐offset constancy and machine‐learning extensions to flag emerging faults automatically ^17^, ^18^, ^19^. The AQA Phase 2 software and test suite is an updated version from that used by Eckhause et al. and provides a customizable EPID‐based QA platform (hosted on Amazon Web Services) that couples a web portal for image upload and automated analysis with the option to import linac service logs in XML format. In this work, AQA EPID results serve primarily as a consistent measurement instrument, aligned with the recent guidance of TG‐330 emphasizing EPID use for relative dosimetry, flood‐field awareness, geometry referenced to the radiation axis, and the value of automation.^20^ In this study, our objective is to investigate the effects of real‐world service events on LINAC beam characteristics as measured by EPID, by quantifying the expected direction and magnitude of changes after photon dose calibration, beam profile steering, and collimator jaw gain–offset calibration and applying an unsupervised, density‐based clustering algorithm (DBSCAN) to identify additional outliers that may indicate undocumented or indirect service impacts.

## 2 MATERIALS AND METHODS

### 2.1 The AQA test suite and linac service logs

The AQA Phase 2 QA suite is comprised of 23 EPID acquisitions delivered using a single DICOM file. The EPID acquisitions are acquired using the MV dosimetry mode setting and uploaded to the AQA website for automatic analysis on a platform hosted on Amazon Web Services. For TrueBeam Linacs, service logs are automatically generated XML records and include 9 broad maintenance categories: Beam Generation Module (BGM) Collimator, Couch, KV detector and source, MV detector, Stand, Supervisor and Xray Imager. The service entries include dosimetry and mechanical calibration events, diagnostic checks, access or workflow entries and routine non‐actionable records. In a similar manner to the DICOM EPID files, the corresponding service logs can be uploaded to the AQA website and displayed at the same timeframe as the Phase2 acquisition data. The AQA workflow does not assume that every service‐log entry is clinically meaningful. Instead, the analysis begins by importing the entire service log history for a given period and then focuses on selected service categories with a plausible relationship to EPID‐measured beam characteristics. This study concentrates on two service node categories. The beam generation module (BGM) category includes the dose calibration and beam steering service events, and the COLLIMATOR category includes Jaw, MLC, and carousel service events. For this study we select beam‐steering events based on service adjustments to the radial or transverse angle current on the steering coils (designated as ANGLE R or ANGLE T) and jaw‐calibration events based on GAIN‐OFFSET service adjustments. Table 1 lists the distribution of Phase 2 EPID QA acquisitions and the service log history for 9 TrueBeam linear accelerators across 6 institutions.

**TABLE 1:** Number of AQA Phase 2 EPID QA acquisitions and corresponding service log history for 9 participating linear accelerators.

LINAC	EPID QA Acquisitions	Total # of Machine Linac Service Events	Beam Generation Module	Collimator	Other/unclassified
1	61	210	161	9	40
2	65	135	73	16	46
3	88	170	82	45	43
4	103	539	405	19	115
5	57	253	160	34	59
6	113	581	455	54	72
7	55	257	195	14	48
8	52	Not available			
9	67	Not available			

*The complete service log history was not available for Linac 8 and 9.

### 2.2 EPID measurement methods

Data were originally collected at a planned frequency of 1–2 times per month, with the end date for data acquisition varying based on staffing availability. The AQA Phase 2 measurements monitor beam output and profile constancy using a 5‐point ROI field analysis and a 20 × 20cm^2^ field size. Each ROI circle is 5 mm in diameter, with the four outer positions equidistant from the beam axis. Axial and transverse symmetry‐based profile constancy were calculated as 100(R−L)/R and 100(T−B)/T, respectively, where R, L, T, and B are the mean EPID responses in the right, left, top, and bottom ROIs. The AQA method samples one paired off‐axis location along each axis and normalizes the difference to a single reference ROI (right or top); therefore, it is more appropriately interpreted as a reference‐normalized profile‐constancy index, rather than a complete TG‐142 symmetry measurement. The ROI position for the center dose and profile symmetry tests is adjusted based on a collimator centering correction to correct for any variation in EPID panel positioning between measurements. The software estimates the collimator centering correction using two 10 × 10 cm^2^ field sizes acquired at collimator angles of 90° and 270°. The X and Y beam center correction is calculated using an average of the four vertical and four horizontal edges respectively and edges are measured by identifying the 50% intensity level. The measured beam center is used as a reference for multiple analyses as a spatial reference point that is independent of EPID panel positioning. For all photon energies and a 20 × 20cm^2^ field size, the AQA analysis includes x‐ and y‐jaw collimator position along with output and profile constancy. Because participating institutions followed different local procedures for EPID recalibration, the AQA consortium did not prescribe a standardized schedule for pixel‐map or flood‐field calibration. The general practice was to recalibrate only when indicated by institutional dosimetry protocols. In addition, TrueBeam service logs do not record pixel‐map and flood‐field calibrations as discrete service‐event categories, so these events were not systematically available for retrospective analysis. When an institution performed EPID recalibration, the event could be entered manually into the AQA event log, enabling comparison with subsequent changes in EPID‐derived QA metrics. The AQA website provides a bulk download function for each participating institution in CSV format. Custom MATLAB and PYTHON routines were developed to extract the relevant measurement data and service logs.

### 2.3 Comparison of EPID‐derived beam output changes

Retrospective machine‐event data were fully available for two linacs (6 and 7) on which the same physicist conducted the AQA measurements. For each eligible 6 MV dose‐calibration event on Linacs 6 and 7, the nearest AQA acquisition before and after calibration was paired with the corresponding pre‐ and post‐calibration ADCL‐traceable ion‐chamber measurements. Percent changes were calculated independently as

(1) $$\Delta \mathrm{EPID}=100\frac{\mathrm{EPI}D_{\mathrm{post}}-\mathrm{EPI}D_{\mathrm{pre}}}{\mathrm{EPI}D_{\mathrm{pre}}}$$

(2) $$\Delta \mathrm{IC}=100\frac{IC_{\mathrm{post}}-IC_{\mathrm{pre}}}{IC_{\mathrm{pre}}}$$

The paired difference, ΔEPID−ΔIC, was then calculated for each event and summarized as the mean difference (bias), standard deviation, and range.

### 2.4 Density‐based clustering (DBSCAN) analysis

We applied DBSCAN to the EPID QA time‐series data to identify atypical measurement changes as candidates for service‐related shifts. DBSCAN identifies locally dense regions as clusters and labels isolated, low‐density points as “noise,” without requiring the number of clusters to be specified in advance ^21^, ^22^, ^23^, ^24^. DBSCAN was applied separately to each one‐dimensional dataset defined by a linear accelerator (LINAC) or EPID metric, including changes between successive EPID acquisitions in center‐axis output, beam symmetry, jaw position, and collimator‐centering correction. The DBSCAN density criterion is controlled by the neighborhood radius, ε (eps), and the minimum number of observations required within an ε ‐neighborhood for an observation to be classified as a core point, min_samples. We selected min_samples = 6 as a conservative setting for these one‐dimensional, modest‐length QA time series. At the typical acquisition frequency of one to two measurements per month, six observations represented approximately 3–6 months of accumulated measurements. The value of ε was selected using the k ‐distance approach, in which the distance from each observation to its k th nearest neighbor was calculated and the resulting distances were sorted to characterize the transition between relatively dense and sparse regions. The neighbor order was set to k=6, corresponding to min_samples = 6, and ε was defined as the 90th percentile of the k ‐distance distribution to provide a reproducible, dataset‐specific selection rule. The 90th‐percentile criterion was a prespecified operational implementation of the k ‐distance method rather than a universal DBSCAN threshold. Conceptually, smaller k (min_samples) values tend to produce larger clusters and fewer noise points, while larger k values require broader neighbor support and may label more points as noise. We therefore selected k = 6 as a balanced outlier screen for longitudinal QA signals.

## 3 RESULTS

### 3.1 EPID center dose output

Figure 1 presents center‐dose CU acquisition histories for two representative linear accelerators from two different institutions, plotted with the corresponding 6MV dose‐calibration events extracted from service logs. These machines were chosen to illustrate typical performance rather than a situation with significant outliers. Using keyword‐based parsing, seven dose calibration events were identified for Linac 3 (Figure 1(a)). For Linac 6, eleven dose calibration events were recorded (Figure 1(b)). Table 2 summarizes the total number of 6MV dose calibration events for Linacs 1–7 and the change in EPID CAX output following a dose calibration event between successive EPID acquisitions. Across the seven linacs, EPID center‐dose constancy showed a consistent post‐calibration decrease relative to baseline (mean SD = −1.10% ± 0.26%; range: −0.73% to −1.54%). Using a two‐sided Welch t‐test, the mean center‐dose change following documented dose‐calibration events differed significantly from the mean change for all remaining non‐calibration events (*p* < 0.05). Figure 2 illustrates the comparison of EPID CAX percent change (ΔEPID) following a 6MV dose calibration event for Linacs 6 and 7 versus the corresponding percent dose change as measured with an ADCL calibrated ion chamber and electrometer (ΔIC). The time‐average paired‐difference between EPID and ion chamber measurement was 0.09% ± 0.79% (*n* = 12, range −1.49% to 1.23%).

**FIGURE 1:**
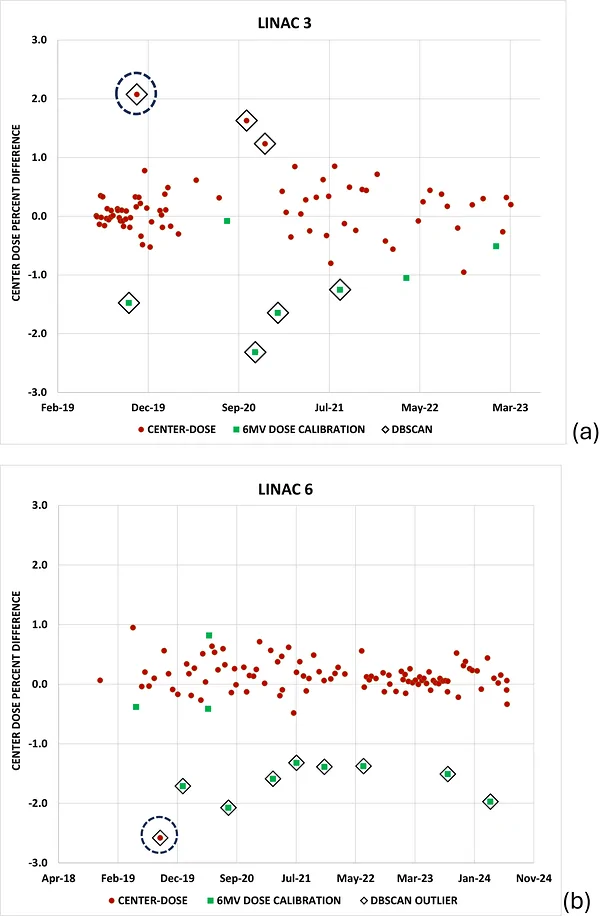
The percent difference change in the center dose output between successive EPID acquisitions, Linac 3 (a) and Linac 6 (b) respectively. The green data squares correspond to 6 MV dose‐calibration events extracted from service logs and the diamonds represent the DBSCAN outlier values.

**TABLE 2:** 6MV dose‐calibration summary for seven participating linear accelerators and the difference between average percent change in the EPID CAX output for QA measurements preceded by a dose calibration versus non‐calibration events. Values are reported as mean (standard deviation).

LINAC	6MV dose calibration events	% change in EPID output (cal events)	% change in EPID output (non‐cal events)	# of CAX Output Measurements	Two‐sided Welch t test *p* < 0.05
1	8	−1.35 (1.00)	0.22 (0.60)	60	YES
2	4	−0.77 (0.46)	0.04 (0.25)	64	YES
3	7	−1.19 (0.74)	0.13 (0.46)	87	YES
4	12	−0.91 (0.90)	0.13 (0.42)	107	YES
5	8	−1.29 (0.74)	0.23 (0.36)	60	YES
6	11	−1.13 (0.81)	0.12 (0.36)	116	YES
7	5	−1.53 (0.65)	0.20 (0.30)	53	YES

**FIGURE 2:**
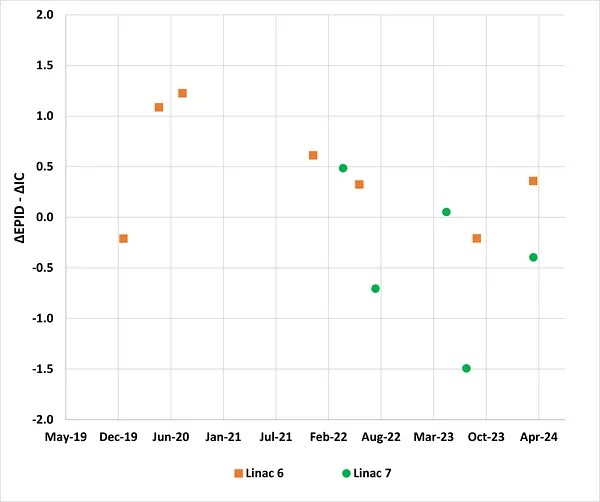
The paired difference (ΔEPID−ΔIC) between EPID and ion chamber for LINAC 6 and 7. ΔEPID represents the percent change for the EPID center‐dose acquisition before and after a 6MV dose calibration event. ΔIC is the corresponding percent change pre‐ and post‐calibration ADCL‐traceable ion‐chamber measurements.

### 3.2 Axial and transverse symmetry

Figure 3 presents axial and transverse symmetry histories for two specific linear accelerators, accompanied by corresponding beam‐steering events extracted from service logs during the same period. One ANGLE R/ANGLE T steering event precedes an observed symmetry shift for Linac 2 at 10FFF beam energy (Figure 3(a) dashed line August 2022) and Linac 7 at 6MV beam energy (Figure 3(b) dashed line September 2022). For Linac 7, the EPID measured an absolute percent change in the axial and transverse symmetry of 1.35% and 0.74% respectively for the acquisition dates of 8/29/22 and 10/1/22. The in‐house daily QA records for Linac 7, measured with an ion chamber‐array system (Sun Nuclear DQA3 check device) document a corresponding percent change in the axial and transverse symmetry of 0.54% and 0.74% for the same dates. Symmetry changes independent of documented ANGLE R/ANGLE T steering events were also observed for Linac 7 as shown by arrows in Figure 3(b). Arrow A corresponds to a 0.95% change in axial beam symmetry as measured with the EPID. The Linac 7 service logs do not list a corresponding beam steering event, and the only documented service event is a carousel‐ion chamber adjustment that occurred between 8/23/22 and 8/29/22. The changes in axial and transverse symmetry highlighted by circle B coincided with annual linac QA measurements. Although beam steering was not performed on either date, a 6 MV dose‐calibration event was logged.

**FIGURE 3:**
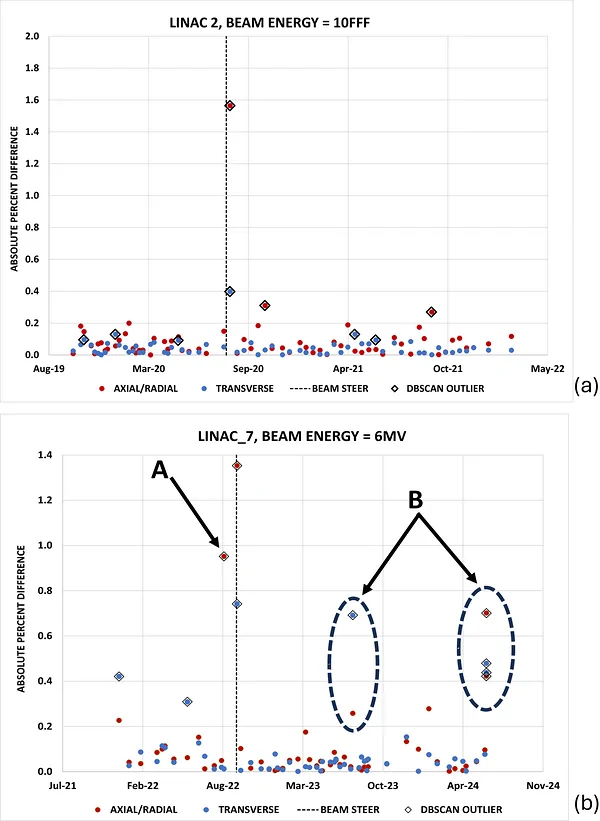
The change in axial and transverse symmetry for Linac 2 operating at 10FFF beam energy (a) and Linac 7 operating at 6MV beam energy (b), plotted as the absolute percent difference between successive EPID QA acquisitions. The dotted line represents a single beam steering event, and the diamonds represent the DBSCAN outlier values. Arrow A highlights a documented carousel/ion chamber adjustment prior to the EPID QA acquisition and arrow B a documented panel flood‐field recalibrations following TG‐51 calibration sessions.

### 3.3 X‐ and Y‐JAW collimator position

Figure 4(a) presents the change in jaw position for Linac 7 between successive EPID QA acquisitions. The service logs for this machine list a single GAIN‐OFFSET calibration event for the Y2 jaw that corresponds to a 1.3 mm change in the Y2 jaw position between the EPID QA acquisitions that straddle the service event (dashed line labeled “Y2‐JAW CALIBRATION” on Jan 2024). DBSCAN cluster analysis identified 15 outlier events including Y2 jaw position change between successive EPID QA acquisitions. One of the outlier events (Arrow A) corresponds to carousel/ion‐chamber adjustments performed between the AQA acquisitions on 8/23/22 and 8/29/22 and represents the same service event identified by the outliers associated with changes in axial and transverse symmetry (Figure 3(b)). Another two outlier events coincided with the dotted line that is the ANGLE‐R beam steering event previously identified in Figure 3. Figure 4(b) presents the change in the total collimator center offset for Linac 7 along with the three common service events: carousel‐ion chamber adjustment (Point A), ANGLE‐R beam steer, and Y2‐jaw calibration. Included on the plot are the DBSCAN outlier events applied to the change in the total offset.

**FIGURE 4:**
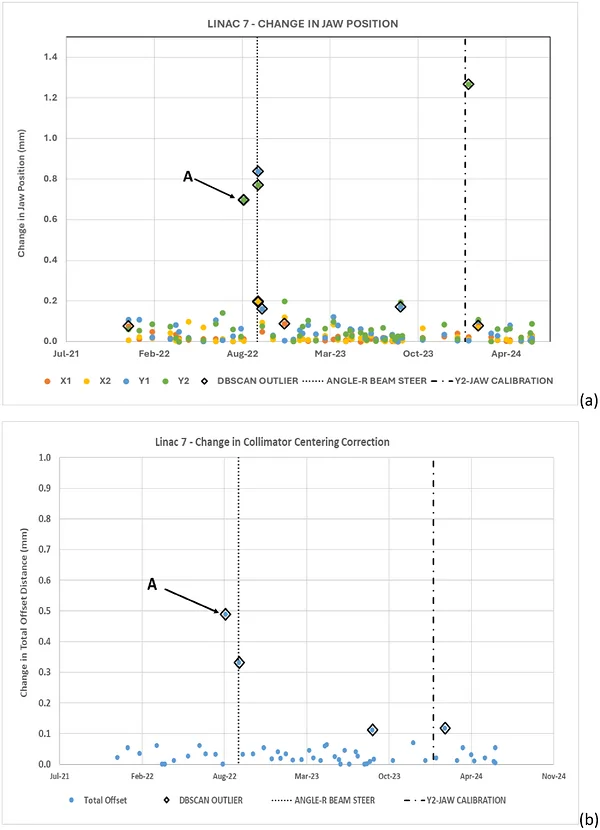
The change in jaw position (a) and the collimator centering offset correction (b) between successive EPID QA acquisitions for AQA Linac 7. The dotted and dashed lines represent an ANGLE‐R beam steering and Y2‐JAW calibration service event respectively. The diamonds represent the DBSCAN outlier analysis applied to the change in the jaw position and the total offset correction. Arrow A highlights a documented carousel/ion chamber adjustment prior to the EPID QA acquisition.

### 3.4 DBSCAN outlier detection

For Linac 3 Figure 1(a), the DBSCAN algorithm identified seven outlier events and four of the outliers correspond to 6MV dose‐calibration events. Of the remaining three outliers only one corresponds to a manually documented service log entry noting a flood‑field and panel output calibration for the 6 MV beam (dashed circle). For Linac 6 Figure 1(b), the DBSCAN algorithm flags a total of nine outlier events; eight correspond to 6MV dose‐calibration events and the remaining outlier value corresponds with multiple maintenance entries documenting linac energy‑switch adjustments prior to the QA measurement marked by the dashed circle. Table 3 summarizes the results when applying the DBSCAN clustering algorithm to the change in EPID CAX output for the nine linacs. There is a wide range for agreement in matching outlier events and calibration events, 100% for Linac 7 (5 of 5) and only 25% for Linac 1 (2 of 8). For the nine linacs, the average change in the EPID center‐dose outlier values was −1.2% ± 0.65% (range −1.96% to −0.25%) versus the average change for the single cluster, non‐outlier values of 0.1% ± 0.01% (range 0.01% to −0.20%). Figure 4(a) presents the DBSCAN outlier analysis for the change in jaw position for Linac 7. The outlier cluster averaged 0.44 mm ± 0.37 mm (*n* = 15, range 0.08–1.27 mm), whereas the non‑outlier cluster averaged 0.03 mm ± 0.02 mm. Figure 5 illustrates the DBSCAN outlier analysis applied to changes in axial and transverse beam symmetry for one representative linac. Of the 14 DBSCAN outlier points identified, 11 occurred on EPID acquisitions that were preceded by a documented beam‐steering–related service event. These included two ANGLE R/ANGLE T adjustments (dotted lines), two Symmetry‐Balance‐Gain calibration adjustments (black circles), and four Gun Grid V/RFDR V adjustments (black squares).

**TABLE 3:** DBSCAN outlier summary for the 9 participating linear accelerators and the comparison of the average percent change in the 6MV EPID CAX output for outlier events versus the non‐outlier cluster. Values are reported as mean (standard deviation).

LINAC	Total dose calibration events	Total DBSCAN outlier points	Matching dose calibration and DBSCAN outlier points	% change in EPID output (outlier)	% change in EPID output (non‐outlier)
1	8	4	2	−0.33 (2.73)	0.03 (0.59)
2	4	3	2	0.51 (1.06)	0.01 (0.25)
3	7	7	4	−0.25 (1.82)	0.05 (0.37)
4	12	9	7	−1.22 (0.92)	0.13 (0.39)
5	8	5	5	−1.62 (0.32)	0.18 (0.43)
6	11	9	8	−1.62 (0.48)	0.14 (0.26)
7	5	5	5	−1.54 (0.65)	0.20 (0.31)
8	—‐	3	—‐	−1.96 (0.37)	0.08 (0.55)
9	—‐	5	—‐	−1.70 (0.52)	0.12 (0.35)

—‐ service logs not available.

**FIGURE 5:**
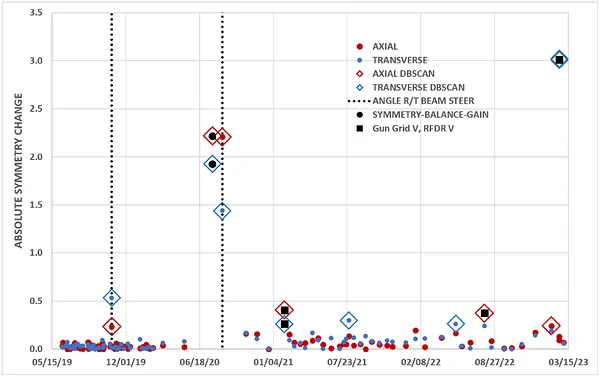
Linac 3 EPID QA acquisition history for the change in axial and transverse beam symmetry and the corresponding DBSCAN outlier values.

## 4 DISCUSSION

### 4.1 General discussion

This multi‐institutional evaluation examines how real‐world maintenance and calibration activities manifest as changes in EPID‐based LINAC constancy metrics when those measurements are interpreted alongside machine service documentation. Although the present analysis is limited to a single manufacturer platform (Varian TrueBeam), largely because of the available consortium infrastructure and service‐log access, the methodology is intentionally independent of the manufacturer's integrated QA application. EPID QA metrics were derived using consortium‐developed software, demonstrating a pathway toward manufacturer‐independent analysis. Importantly, service‐log access is not required for AQA's core image analysis and longitudinal trending of output, beam‐profile constancy, jaw position, and other EPID‐derived metrics. When service logs are unavailable, DBSCAN and similar unsupervised methods can still flag atypical measurements for investigation, although the cause of an observed change cannot be attributed to a specific maintenance or calibration event without additional documentation. The core workflow, standardized EPID acquisition, consistent metric extraction, longitudinal trending, and unsupervised outlier triage, should therefore be adaptable to other manufacturers, machine models, and clinical environments, provided physicists have access to QA‐grade EPID images and associated metadata. Event‐level interpretation would additionally require access to a searchable service and calibration history, whether supplied by the manufacturer or maintained locally. Previous MPC studies have demonstrated sensitivity to real‐world faults and the value of longitudinal trend analysis ^14^, ^15^. The added contribution of the present approach is the systematic linkage of manufacturer‐independent AQA measurements and DBSCAN‐flagged changes to specific service events, providing additional context for interpreting QA trends and selecting targeted post‐service measurements. Concurrent MPC results were not analyzed in this study and therefore, the fault‐detection benefit of AQA beyond MPC remains unestablished. Prospective comparisons using matched pre‐ and post‐service acquisitions, documented calibration histories, and independent dosimetric and geometric measurements are needed to determine whether service‐linked AQA review improves interpretation or follow‐up decisions beyond MPC alone.

### 4.2 EPID center dose output, beam symmetry and collimator jaw calibration

For linacs 1–7 the average 6MV post‐calibration decrease is consistent with a scenario in which output had drifted high relative to the clinical reference before the calibration and was adjusted back toward nominal during the TG‐51/output calibration procedure. Comparing the EPID output measurements versus ion chamber calibration records for two linacs, the average percent difference between EPID CAX output change and ion chamber measurement (mean ± SD = 0.09% ± 0.79%) is consistent with prior EPID–ion chamber photon output comparisons ^5^, ^11^, ^15^. Correlations were observed between documented beam steering service events and symmetry shifts detected by EPID measurements. Adjustments involving radial and transverse angle current on the steering coils (ANGLE R or ANGLE T) appear particularly relevant. For a representative linac (Linac 7) and a documented ANGLE R/ANGLE T steering event, the EPID agreed with daily array measurements within 0.8% across the corresponding dates. This agreement is consistent with the analysis of Sun et al., who validated EPID‐derived symmetry against a 2D ion‐chamber array with agreement within ± 0.5% (crossline) and ± 1.2% (inline) across deliberate symmetry detunes of 2%–4% ^5^. Figure 3(b) highlights the symmetry changes not associated with ANGLE R/ANGLE T steering events for Linac 7. In‐house records for this linac confirmed both TG‐51 absolute dose calibration and EPID panel recalibration on those dates, so the observed symmetry change may reasonably be attributed to the flood‐field recalibration, which can affect the apparent EPID symmetry response, rather than to the dose calibration itself. The observed changes in the Y1‐ and Y2‐jaw position following carousel ion‐chamber and beam steering adjustments are most plausibly measurement artifacts rather than physical changes in jaw position. These service events act on the effective focal‐spot position and beam centering and not the mechanical position of the jaws. Because the jaw positions are inferred from the radiation field edge, any steering‐induced focal‐spot migration or beam‐axis redefinition will shift the apparent field edge even if the physical jaws have not moved.

### 4.3 DBSCAN outlier detection

The DBSCAN clustering algorithm was evaluated to flag noise clusters as candidates for atypical, service‐related shifts in the EPID measurements and provide preliminary evidence on the use of an unsupervised clustering algorithm to detect QA measurement outliers. Across output, symmetry, and jaw‐position metrics, DBSCAN highlighted atypical measurements that were often temporally associated with documented service activity. For center‐dose output (Figure 1), DBSCAN identified outliers near dose‐calibration events and flagged additional output changes associated with manually documented flood‐field/panel‐output calibration or energy‐switch maintenance. For beam symmetry (Figures 3 and 5), most outliers were preceded by beam steering related service entries, including ANGLE R/T, Symmetry‐Balance‐Gain, and Gun Grid V/RFDR adjustments. However, some symmetry outliers were associated with non‐steering activities such as carousel/ion‐chamber work or panel flood‐field recalibration, suggesting that profile‐constancy changes may reflect multiple service or detector‐calibration mechanisms. For jaw‐position and collimator‐centering metrics (Figure 4), DBSCAN identified discrete deviations temporally associated with the documented Y2 jaw gain‐offset calibration and with the same carousel/ion‐chamber event noted in the symmetry analysis. Together, these findings support DBSCAN as a practical method for screening longitudinal EPID QA data and prioritizing atypical measurements for physicist review.

### 4.4 Limitations

Several limitations should be considered when interpreting these findings. EPID acquisition frequency was not standardized across participating centers, and measurements were not prospectively timed immediately before and after service events. Therefore, EPID changes may be linked in time to service activity, but this study cannot prove that the service event caused the change. In addition, we could not calculate a true sensitivity rate because it is unknown whether service events that should have affected LINAC performance produced no detectable change in the EPID metrics available for analysis. Documentation of EPID flood‐field and panel output recalibrations was also inconsistent and not systematically incorporated into routine service logs, limiting attribution in some cases. Future studies should use standardized pre‐ and post‐service EPID acquisitions, centralized documentation of EPID recalibrations, and the commissioning framework described in the 2025 IPEM MIQC guidance, including detector commissioning, repeatability and reproducibility testing, baseline/action‐level definition, concordance with existing QC, and sensitivity testing ^25^. The limited independent comparisons in this study, ion‐chamber calibration measurements on two linacs and one detector‐array symmetry comparison, do not establish the detection performance of the complete AQA–service‐event workflow. The consortium study by Critchfield et al. ^26^ provides a framework for systematic sensitivity and concordance testing against established reference measurements, but validation of selected AQA tests does not replace independent verification of individual service‐associated changes. Future studies should extend reference comparisons across more linacs, both around service events and during routine operation, including acquisitions not flagged by DBSCAN. Such a design would support assessment of measurement agreement and detected or missed changes, strengthening the clinical interpretation of EPID‐derived findings. The DBSCAN analysis also has important limitations. The selected parameters, including min_samples/k = 6 and the use of the 90th‐percentile k‐distance to define ε, were chosen as a reproducible and conservative approach for longitudinal QA data, but they were not optimized against a prospective ground truth. Different choices of k and ε could change the number of measurements labeled as noise and therefore alter the apparent sensitivity of the outlier‐detection workflow. Future work should evaluate parameter robustness by comparing multiple k/ε settings against known service events and independent dosimetric or geometric measurements. Finally, DBSCAN‐labeled noise points should not be treated as independent groups for conventional inferential testing against the remaining non‐noise points. Because the noise labels are assigned from the same EPID values being compared, tests such as Welch's t‐test between noise and non‐noise measurements would be circular and may simply confirm the clustering definition rather than demonstrate an independent effect. Accordingly, DBSCAN should be interpreted as a triage tool that identifies atypical EPID measurements for review, not as proof of a service‐related fault. Future analyses may incorporate internal cluster‐validation approaches designed for density‐based methods with noise, such as DISCO, which explicitly evaluates noise assignments and is defined for cases such as a single cluster plus noise.

## 5 CONCLUSIONS

Using the AQA Phase 2 EPID suite as a standardized, manufacturer‐independent analysis method and time‐aligning its results with TrueBeam service logs, this multi‐institutional study characterized EPID‐measured changes associated with common real‐world service events. EPID center‐dose decreased following documented 6 MV dose calibrations across all seven evaluated linacs; however, independent ADCL‐traceable ion‐chamber comparison was available for only two linacs and 12 calibration events. The observed symmetry changes following beam‐steering activities and the changes in EPID‐derived jaw position and collimator centering following jaw calibrations were identified from longitudinal EPID measurements aligned with service records and were not independently verified using separate profile or geometric measurement systems. Similarly, DBSCAN provided an EPID‐internal method for flagging atypical measurements for review rather than independent confirmation of a machine fault. These results therefore identify service events associated with measurable changes in EPID constancy metrics and support targeted post‐service QA, but they do not establish causality or independently validate every observed beam or geometric change. Prospective studies incorporating standardized EPID acquisitions immediately before and after service, together with independent dosimetric and geometric measurements, are needed to confirm these associations and define their clinical significance.

## Author Contribution

John DeMarco: Initial Investigation, Methodology, Data Collection, Interpretation of Data, Writing‐Original Draft, Michael Barnes: Initial Investigation, Methodology, Data Collection, Interpretation of Data, Writing‐Review & Editing, Hania Al‐Hallaq: Initial Investigation, Methodology, Data Collection, Interpretation of Data, Writing‐Review & Editing, Seng Boh Lim: Methodology, Interpretation of Data, Writing‐Review & Editing, Lana Critchfield: Methodology, Writing‐Review & Editing, Cory Knill: Methodology, Writing‐Review & Editing, Karl Farrey: Data Collection, Interpretation of Data, Writing‐Review & Editing, Timothy Ritter: Methodology, Writing‐Review & Editing, Nilendu Gupta: Methodology, Writing‐Review & Editing, Richard Popple: Methodology, Writing‐Review & Editing, Justin Mikell: Initial Investigation, Methodology, Data Collection, Interpretation of Data, Writing‐Review & Editing, Jim Irrer: Initial Investigation, Methodology, Mario Perez: Initial Investigation, Methodology, Data Collection, Interpretation of Data, Writing‐Review & Editing, Jean Moran: Initial Investigation, Methodology, Interpretation of Data, Writing‐Review & Editing

## CONFLICT OF INTEREST STATEMENT

The authors disclose that this study was supported in part by a grant from Varian

## FUNDING INFORMATION

The authors disclose that this study was supported in part by a grant from Varian.

## ETHICS OR IRB COMPLIANCE STATEMENT

None. This study does not require IRB or ethics approval

## Data Availability

This manuscript submission does not require shared data.
